# Supplementary material for: Altered microRNA Transcriptome in Cultured Human Liver Cells upon Infection with Ebola Virus
Source: Int J Mol Sci. 2021 Apr 6;22(7):3792. doi: 10.3390/ijms22073792 (PMC8038836; doi:10.3390/ijms22073792)
Supplement: Supplementary file 1 [file ijms-22-03792-s001.zip › Supplementary_File/C_ GO_Analysis_Results/16-30nt_go_Mayinga-24h-Huh7_vs_Control-24h-Huh7_up.mature_mirna_targets/GeneOntology_FoldEnrichment.pdf]

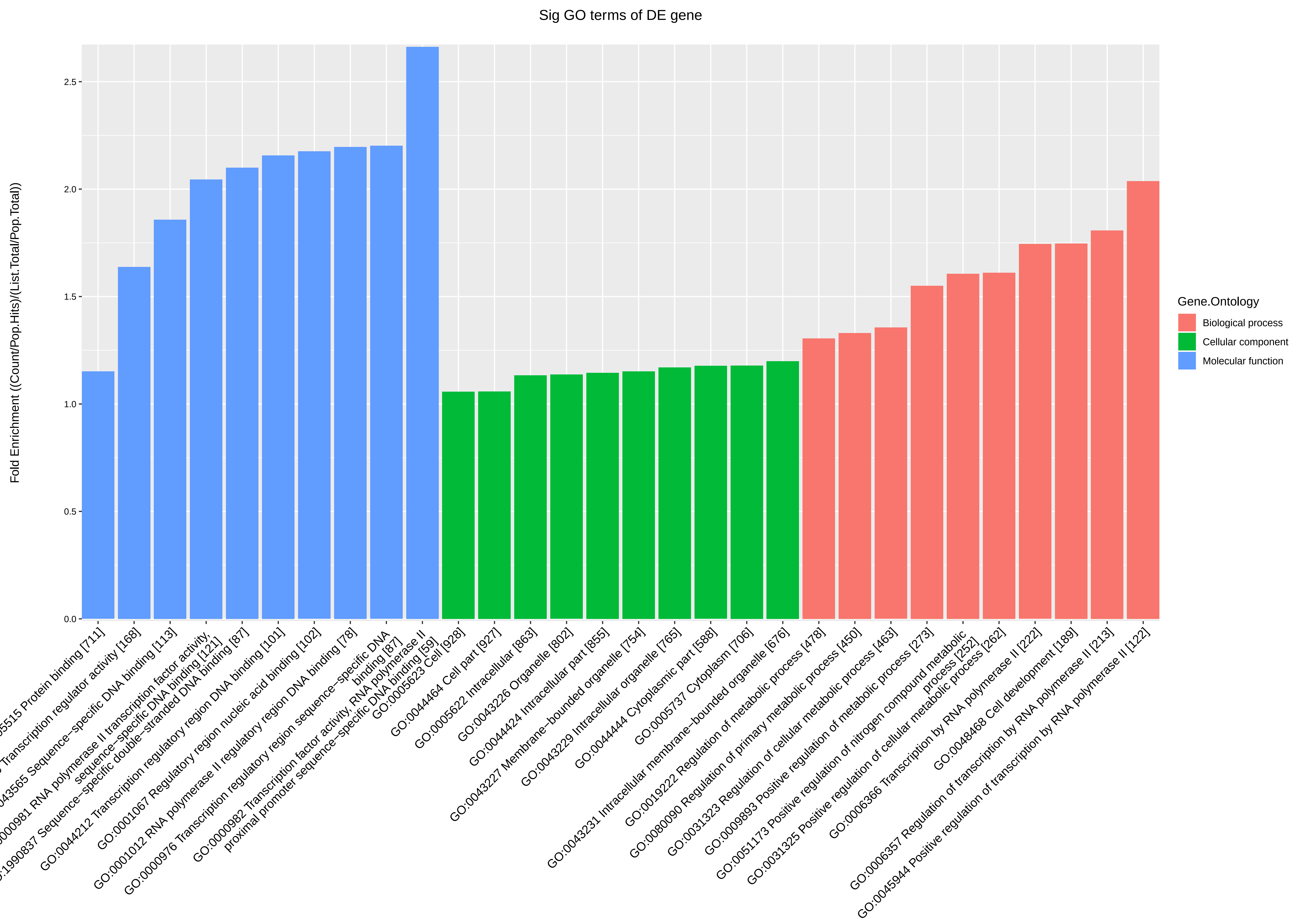
Sig GO terms of DE gene
Fold Enrichment ((Count/Pop.Hits)/(List.Total/Pop.Total))
0.0
0.5
1.0
1.5
2.0
2.5
Gene.Ontology
Biological process
Cellular component
Molecular function
GO:0005515 Protein binding [711]
GO:0140110 Transcription regulator activity [168]
GO:0043565 Sequence−specific DNA binding [113]
GO:0000981 RNA polymerase II transcription factor activity, sequence−specific DNA binding [121]
GO:1990837 Sequence−specific double−stranded DNA binding [87]
GO:0044212 Transcription regulatory region DNA binding [101]
GO:0001067 Regulatory region nucleic acid binding [102]
GO:0001012 RNA polymerase II regulatory region DNA binding [78]
GO:0000976 Transcription regulatory region sequence−specific DNA binding [87]
GO:0000982 Transcription factor activity, RNA polymerase II proximal promoter sequence−specific DNA binding [59]
GO:0005623 Cell [928]
GO:0044464 Cell part [927]
GO:0005622 Intracellular [863]
GO:0043226 Organelle [802]
GO:0044424 Intracellular part [855]
GO:0043227 Membrane−bounded organelle [754]
GO:0043229 Intracellular organelle [765]
GO:0044444 Cytoplasmic part [588]
GO:0005737 Cytoplasm [706]
GO:0043231 Intracellular membrane−bounded organelle [676]
GO:0019222 Regulation of metabolic process [478]
GO:0080090 Regulation of primary metabolic process [450]
GO:0031323 Regulation of cellular metabolic process [463]
GO:0009893 Positive regulation of metabolic process [273]
GO:0051173 Positive regulation of nitrogen compound metabolic process [252]
GO:0031325 Positive regulation of cellular metabolic process [262]
GO:0006366 Transcription by RNA polymerase II [222]
GO:0048468 Cell development [189]
GO:0006357 Regulation of transcription by RNA polymerase II [213]
GO:0045944 Positive regulation of transcription by RNA polymerase II [122]
